# Low intensity focused ultrasound (LOFU) modulates unfolded protein response and sensitizes prostate cancer to 17AAG

**DOI:** 10.18632/oncoscience.48

**Published:** 2014-06-03

**Authors:** Subhrajit Saha, Payel Bhanja, Ari Partanen, Wei Zhang, Laibin Liu, Wolfgang Tomé, Chandan Guha

**Affiliations:** ^1^ Department of Radiation Oncology, Albert Einstein College of Medicine, Bronx, New York, USA; ^2^ Philips Healthcare, Cleveland, OH, USA; ^3^ Department of Pathology, Albert Einstein College of Medicine, Bronx, New York, USA; ^4^ Montefiore Medical Center, New York, NY, USA

**Keywords:** Prostate Cancer, 17AAG, Focused Ultrasound, UPR, ER stress

## Abstract

The hypoxic tumor microenvironment generates oxidative Endoplasmic Reticulum (ER) stress, resulting in protein misfolding and unfolded protein response (UPR). UPR induces several molecular chaperones including heat-shock protein 90 (HSP90), which corrects protein misfolding and improves survival of cancer cells and resistance to tumoricidal therapy although prolonged activation of UPR induces cell death. The HSP90 inhibitor, 17AAG, has shown promise against various solid tumors, including prostate cancer (PC). However, therapeutic doses of 17AAG elicit systemic toxicity. In this manuscript, we describe a new paradigm where the combination therapy of a non-ablative and non-invasive low energy focused ultrasound (LOFU) and a non-toxic, low dose 17AAG causes synthetic lethality and significant tumoricidal effects in mouse and human PC xenografts. LOFU induces ER stress and UPR in tumor cells without inducing cell death. Treatment with a non-toxic dose of 17AAG further increased ER stress in LOFU treated PC and switch UPR from a cytoprotective to an apoptotic response in tumors resulting significant induction of apoptosis and tumor growth retardation. These observations suggest that LOFU-induced ER stress makes the ultrasound-treated tumors more susceptible to chemotherapeutic agents, such as 17AAG. Thus, a novel therapy of LOFU-induced chemosensitization may be designed for locally advanced and recurrent tumors.

## INTRODUCTION

Therapeutic ultrasound is being developed as an image-guided ablative treatment for solid tumors. High-intensity focused ultrasound (HIFU) delivers sonic energy to a small, well-defined target region in malignant tissue, causing a rapid rise in tissue temperature exceeding 80-90^o^C, thereby inducing instantaneous coagulative necrosis at the focal point. HIFU is quickly emerging as an effective image-guided, minimally invasive treatment modality for solid tumors, including prostate cancer (PC) [1, 2]. The biological effects of therapeutic ultrasound results from both thermal and non-thermal/mechanical bioeffects, which arise from complex interactions of propagating ultrasound waves with target tissue[3]. Thermal effects are due to ultrasound absorption and conversion to heat through vibrational excitation of tissue, leading to localized temperature elevation. Mechanical bioeffects that are unique to HIFU include acoustic radiation forces and acoustic cavitation[4, 5]. HIFU may pose a risk to normal tissues, e.g., bones, blood vessels, and nerves adjacent to target malignant tissue, due to rapid temperature elevation leading to nearly instantaneous thermal coagulation at high acoustic intensities[5]. Furthermore, bubble activity mediated acoustic cavitation may be induced at high acoustic pressure levels, leading to locally induced stress and high energy release, possibly resulting in and assisting thermal coagulation[5]. The biological outcome of ultrasound therapy can be controlled by adjusting the ultrasound exposure parameters, including ultrasound frequency, acoustic output power, duty cycle, exposure duration, and focal point characteristics. Low *in situ* acoustic pressures and intensities as well as low total absorbed energy in target tissue may induce low-level mechanical stress and mild hyperthermia (40-45**°**C), possibly resulting in cell membrane perturbation without causing significant thermal injury or cell death. The biological effects of low energy focused ultrasound (LOFU) in tissues have not been studied well. We hypothesized that the mechanical and thermal energies deposited by LOFU at tissues in focal point would induce misfolding of newly synthesized proteins in the ER, thereby increasing ER stress and UPR in tumor cells.

Tumor cells experience a variety of cytotoxic and genotoxic stresses, such as hypoxia, low pH, and low nutrients during the course of tumor progression. These conditions, coupled with the higher rate of protein synthesis in tumor cells, result in disruption of protein folding and maturation of secretory and membrane proteins. The accumulation of misfolded proteins in the endoplasmic reticulum (ER) produces a stress response, which induces heat shock proteins (HSPs) to initiate cytoprotective measures to correct protein misfolding and restore normal ER function [6-8]. These responses are collectively known as unfolded protein response (UPR). If the correction machinery fails to control erroneous protein burden, resulting in prolonged activation of the UPR, the cytoprotective response switches to initiate programmed cell death or apoptosis. Thus, the signaling cascade of UPR involves both cytoprotective and apoptotic/cell death pathways.

Recent data suggest that UPR may play a role in protecting transformed cancer cells from the inadequate environment that exists prior to vascularization and therefore contributes to tumor growth and survival [8]. These cytoprotective signaling also contribute to resistance to chemotherapeutic agents. Cell culture studies demonstrated that modulation of UPR by pharmacological agents could alter the sensitivity of tumor cells, making them either more sensitive in some cases or more resistant in others to chemotherapy [9]. The HSP90 molecular chaperone contributes to the UPR-mediated chemoresistance and has emerged as one of the most exciting targets for cancer drug development. The HSP90 inhibitor, 17AAG, has shown promise against various solid tumors, including prostate cancer, by increasing apoptosis of tumor cells [10]. However, therapeutic doses of 17AAG elicit hematopoeitic, hepatic, and gall bladder toxicity, which significantly reduce its effectiveness due to these dose-limiting toxicities [11]. The goal of our experiments was to increase the burden of misfolded proteins in the ER and prolong ER stress in tumor cells, by combining LOFU with a HSP90 inhibitor, e.g., 17AAG, thereby switching the prosurvival UPR to the apoptotic pathway. In the present study, we demonstrate for the first time that non-ablative, non-cavitational LOFU can induce UPR in murine and human prostate cancer cells and sensitize them to nontoxic, low doses of 17AAG.

## RESULTS

### Treatment schema and toxicity of LOFU and 17AAG therapy

**Figure 1:**
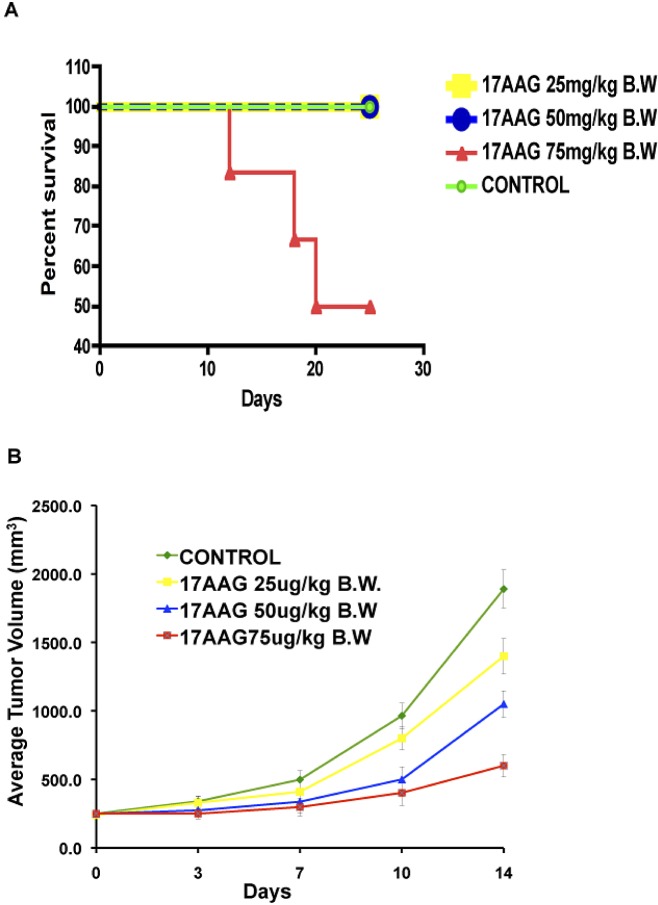
High dose 17AAG reduces tumor growth in C57Bl6 mice but causes mortality from chemotoxicity Systemic administration of 17AAG showed significant effect on tumor growth with higher doses (50-75 mg/kg of body weight) (p<0.003). 50% mice were died receiving 17AAG at a dose of 75mg/kg of body weight within 25 days post-treatment.

For each grid location, LOFU was administered for 1.5 seconds at 100% duty cycle, acoustic power of 3 W, and using ultrasound frequency of 1 MHz. This protocol yielded an approximate *in situ* spatial-peak temporal-average acoustic intensity of 270 W/cm^2^, resulting in estimated average intra-tumoral temperature elevation of 3.2 °C. Post-treatment, there were no signs of normal tissue toxicity such as alopecia, thermal damage, or skin wounds. Preclinical pharmacokinetic [17] studies in mice showed 17AAG to widely distribute [18] and undergo extensive hepatic metabolism [11]. Systemic administration of 17AAG is known to be associated with significant hepatotoxicity, characterized by increases in transaminases and bile acids, and drug-related histopathologic lesions in the gallbladder, common bile duct, and gastrointestinal tract [11]. Therefore, we determined the dose of 17AAG that was nontoxic for our therapy. C57Bl6 mice were treated with intraperitoneal injections of 17AAG (25 – 75 mg/kg body weight) three times a week. Control mice were injected with equal volume of the vehicle DMSO, which was used to solubilize 17AAG. Although higher doses of 17AAG (50-75 mg/kg of body weight) treatment achieved significant tumor growth retardation compared to untreated control (untreated tumor, 1879±98.65 mm^3^ versus 17AAG 75 mg/kg b.w., 485±24.25 mm^3^, p<0.003 and 50 mg/kg b.w., 964 mm^3^, p<0.007, respectively; Figure 1B), Kaplan Meier survival analysis showed death in 50% of mice after 21 days of treatment with a dose of 75 mg/kg of body weight (Figure 1A).

A low dose of 17AAG that was found to be nontoxic was 25 mg/kg in C57Bl/6 mice and 14 mg/kg in Balb/c nude mice. Thus, these dose levels were selected for the current study. The goal was to combine two therapies that are nontoxic, albeit subtherapeutic, and examine whether the combination can be therapeutic.

### LOFU induces the cytoprotective pathways of UPR

**Figure 2:**
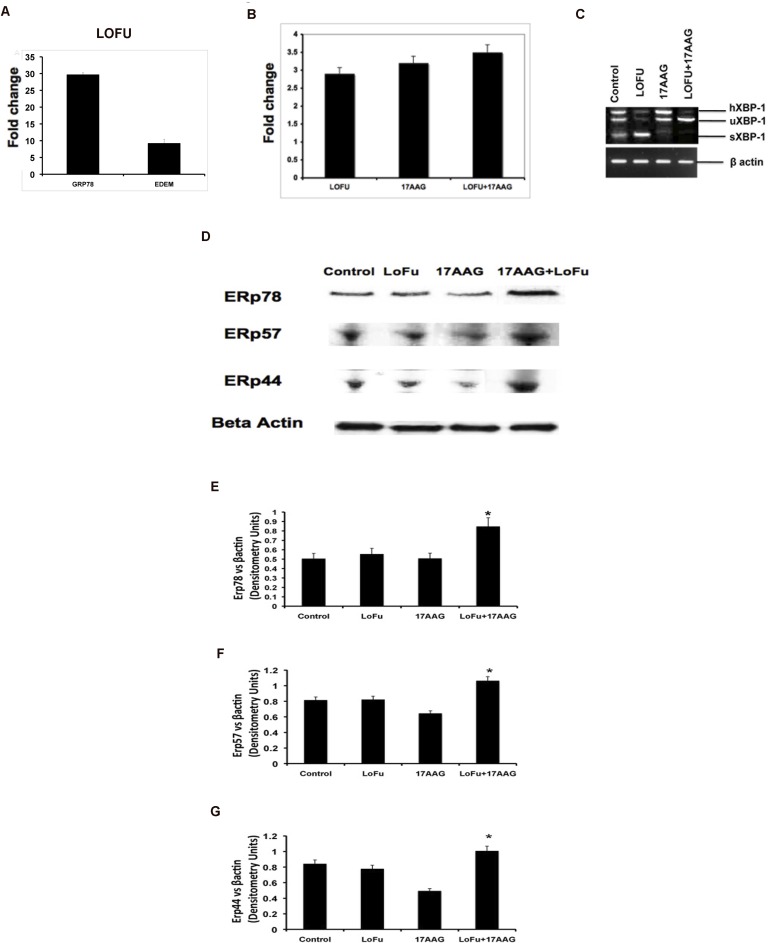
LOFU induces UPR A. LOFU increases the expression of Bip/Grp78 and EDEM mRNAs. Real Time-PCR analysis of RNA isolated from LOFU-treated RM1 tumors showed 29.73±0.56 fold increase in Bip/Grp78 and 9.27±1.18 fold increase in EDEM mRNA level compared to untreated control. B. LOFU increases the expression of IRE1α mRNA by 2.8±0.4 folds. Real Time-PCR analysis demonstrates that LOFU induced increase in the IRE1α expression did not alter with the 17AAG treatment. C. LOFU induced the splicing of XBP1 mRNA. 17AAG treatment inhibits the splicing of XBP1. XBP1s, XBP1h, and XBP1u denote the spliced, hybrid, and un-spliced forms of XBP1, respectively. D-G. LOFU+17AAG combination therapy prolongs ER stress in RM1 tumor cells. Western blot and bar chart showing that the expression of ERP78 (D & E), ERP57 (D & F), and ERp44 (D & G) proteins was induced in combination treatment group.

Misfolded proteins are recognized by ER chaperones, such as BiP/GRP78 protein. Once BiP/GRP78 binds to the misfolded proteins, the ER resident trans-membrane protein kinases, such as, IRE1α are activated. IRE1α has an endoribunuclease activity that is responsible for splicing of XBP1 mRNA. Subsequently, the XBP1 protein transcriptionally activates the expression of various prosurvival genes, including HSPs, thereby promoting cell survival [7, 19]. In order to determine whether LOFU induces UPR, we isolated RNA from PC tumors, 24 hours after the completion of LOFU treatment, and performed qRT-PCR to quantitate the mRNA expression of BiP/GRP78, EDEM and IRE1a. LOFU induced the mRNA levels of Bip/Grp78 (29.73±0.56), EDEM (9.27±1.18) and IRE1α (2.8±0.4) in the tumor tissue, compared to untreated controls (Figure 2A-B). Treatment with 17AAG did not increase IRE1 mRNA expression over the levels achieved after LOFU treatment. Since XBP1 splicing is an indicator of the induction of the cytoprotective pathways during UPR, we quantitated the expression of spliced XBP1 mRNA by RT-PCR after LOFU+17AAG treatment. LOFU induced the splicing of XBP1, while treatment with 17AAG suppressed XBP1 splicing after LOFU treatment (Figure 2C), indicating that LOFU induces the cytoprotective pathways of UPR, while combination treatment of LOFU+17AAG inhibits it.

### Combination treatment of LOFU+17AAG amplifies ER stress

Accumulation of misfolded proteins in the ER induces a stress response with induction of chaperone proteins that help in correction of protein misfolding. To detect the level of ER stress, the expression levels of ER chaperones, ERp44, ERp57, and ERp72 were quantitated among different treatment groups. ERp44 is responsible for oxidative protein folding [20]. ERp57 is an ER resident thiol disulfide oxidoreductase [21] while Erp72 is a disulfide isomerase. All these proteins participate in the protein folding machinery of the ER. Compared to tumor tissues from animals that received no treatment or LOFU or 17AAG alone, immunoblot analysis demonstrated a significant increase in the expression of ERp78 (p<0.03, Figure 2D & E), ERp44 (p<0.05, Figure 2D & G), and ERp57 (p<0.04, Figure 2D & F) protein levels in tumor tissues following combination treatment with LOFU+17AAG. This suggests that 17AAG mediated inhibition of HSP90 may increase the unfolded protein burden in the ER, thereby prolonging ER stress.

### LOFU+17AAG activates pro-apoptotic pathways of UPR and induces apoptosis in mouse and human prostate cancer tissues

**Figure 3:**
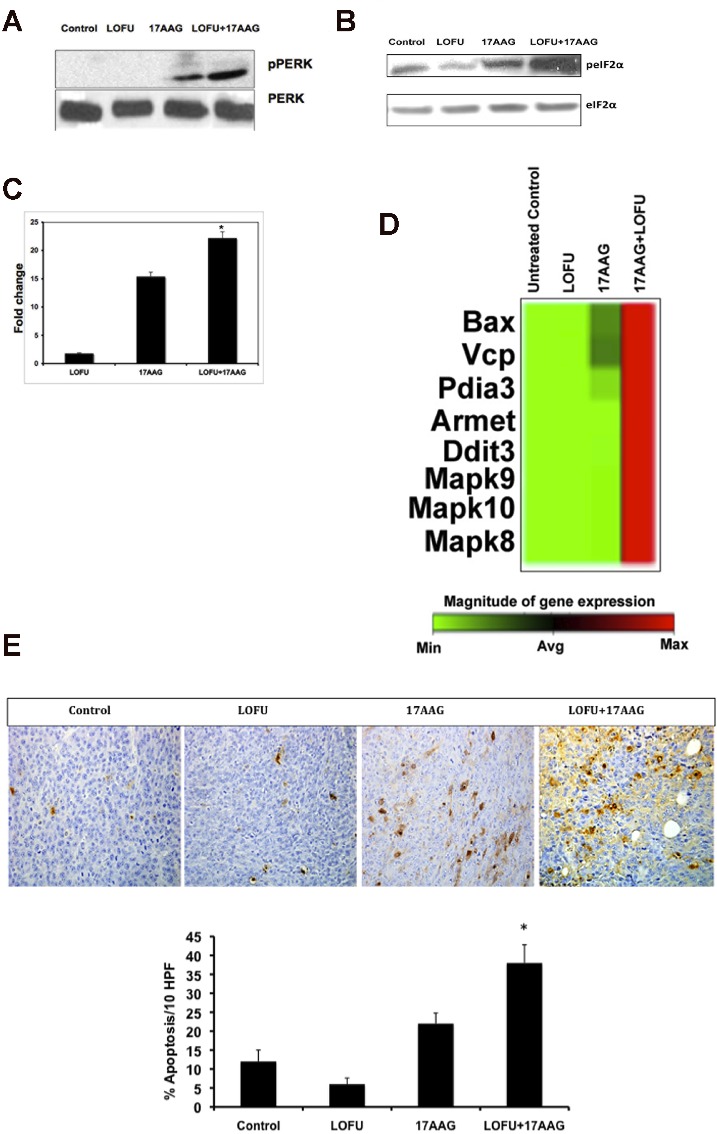
LOFU+17AAG activates pro-apoptotic pathways of UPR and induces apoptosis in tumor cells. A & B Western blot of pPERK (A) and peIF2a (B). LOFU+17AAG activates PERK by phosphorylation of PERK (pPERK), which further induces the phosphorylation of eIF2α phosphorylation (peIF2α). C. Real Time-PCR analysis of CHOP mRNA. There was a 25±1.3-fold increase in CHOP transcript in LOFU+17AAG treated group, compared to control. D. Real Time-PCR array of RNA isolated from LOFU+17AAG-treated tumors. Heat map analysis showed that LOFU+17AAG treatment group increased the transcript level of apoptotic genes several folds compared to untreated control or LOFU groups. E. TUNEL staining. Immunohistochemical staining showed predominantly tunel positive cells in LOFU+17AAG treatment group, compared to control or LOFU group. Note that 17AAG alone also induced apoptosis in tumor tissue that was augmented by LOFU.

ER stress activates the three arms of UPR at the same time, thereby producing antagonistic cytoprotective and apoptotic signals at the same time. The fate of the cell depends upon the ability of its protein correction machinery to lower the ER stress, thereby attenuating the UPR. If ER stress persists, the cytoprotective pathways are eventually overwhelmed with the chronic activation of PERK-mediated apoptotic pathways causing cellular demise. Since phosphorylation of PERK at Thr980 serves as a marker for its activation status, we performed immunoblot analysis that showed a significant increase in pPERK levels in tumor tissue following treatment with 17AAG (Figure 3A). Phosphorylated PERK levels were absent in untreated and LOFU-treated tumors. However, combination treatment of LOFU+17AAG exhibited the highest levels of PERK phosphorylation (Figure 3A).

Since prolonged PERK activation attenuates protein synthesis in response to ER stress through the phosphorylation of translation initiation factor eIF2α at serine 51, we determined the levels of phosphorylated eIF2α. Treatment of RM1 tumors with 17AAG induced phosphorylation of eIF2α over the basal levels in untreated controls. LOFU treatment resulted in marginal reduction of phosphorylated eIF2α levels. However, the highest levels of phosphorylated eIF2α were seen in tumors that received combination treatment with LOFU+17AAG (Figure 3B), corroborating with highest activation of PERK phosphorylation in these tumors compared to other groups.

Although phosphorylated eIF2α decreases the translation of most cellular proteins, including pro-survival and anti-apoptotic proteins, it increases the translation of a transcription factor, ATF4 that is responsible for inducing the transcription of pro-apoptotic genes, such as, CCAAT/enhancer-binding protein homologous protein (CHOP), thereby preparing the cell for programmed cell death in case the misfolded proteins are not repaired and ER stress persists [6]. As expected, LOFU treatment failed to induce CHOP levels (1.6±0.7 fold) over untreated controls. In contrast, treatment with 17AAG alone induced CHOP transcript levels to 14.8±2 fold, which was further increased to 25±1.3 fold (p<0.006) in the combination treatment group of LOFU+17AAG, compared to untreated controls (Figure 3C).

In order to examine whether downstream apoptotic genes are expressed following CHOP induction by the combination therapy of LOFU+17AAG, we performed a mouse UPR qRT-PCR Array on total RNA isolated from tumor tissues of various treatment groups. Heatmap analysis demonstrated that pro-apoptotic target genes, such as Bax, Vcp, Pdia3, Armet, Ddit3, Mapk8, Mapk9, and Mapk10 were induced several folds following combination therapy with LOFU+17AAG compared to untreated controls (Figure 3D). There was minimal induction of pro-apoptotic genes upon treatment with LOFU alone or 17AAG alone. This result indicates that the combination therapy of LOFU+17AAG activates PERK, induces CHOP, and switches on the pro-apoptotic pathway of the UPR. Indeed, TUNEL staining demonstrated that LOFU induced minimal apoptosis over untreated controls. Treatment with 17AAG induced significant apoptosis in prostate tumors, which was further increased by LOFU (p<0.004) (Figure 3E). Thus, 17AAG-mediated inhibition of HSP90 and activation of CHOP by the combination of LOFU+17AAG switched on apoptotic cell death of prostate tumors.

### LOFU+17AAG inhibits Chaperone Mediated Autophagy (CMA) in tumor cells

**Figure 4:**
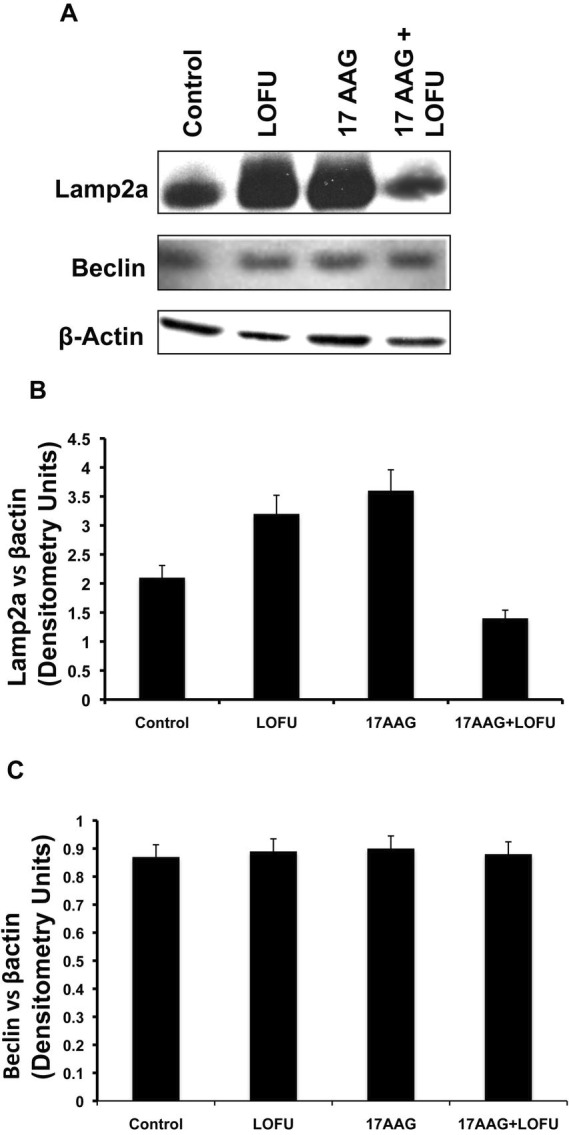
LOFU+17AAG treatment inhibits Chaperone Mediated Autophagy (CMA) in RM1 tumor cells **(A & B)** Immunoblot analysis showed several fold down-regulation of SMA marker LAMP2a expression level in combination treatment group. Treatment with either LOFU or 17AAG up-regulates the LAMP2a expression level. **(A & C)** Combination treatment of LOFU and 17AAG did not alter the expression level of Beclin, a macroautophagy marker.

**Figure 5:**
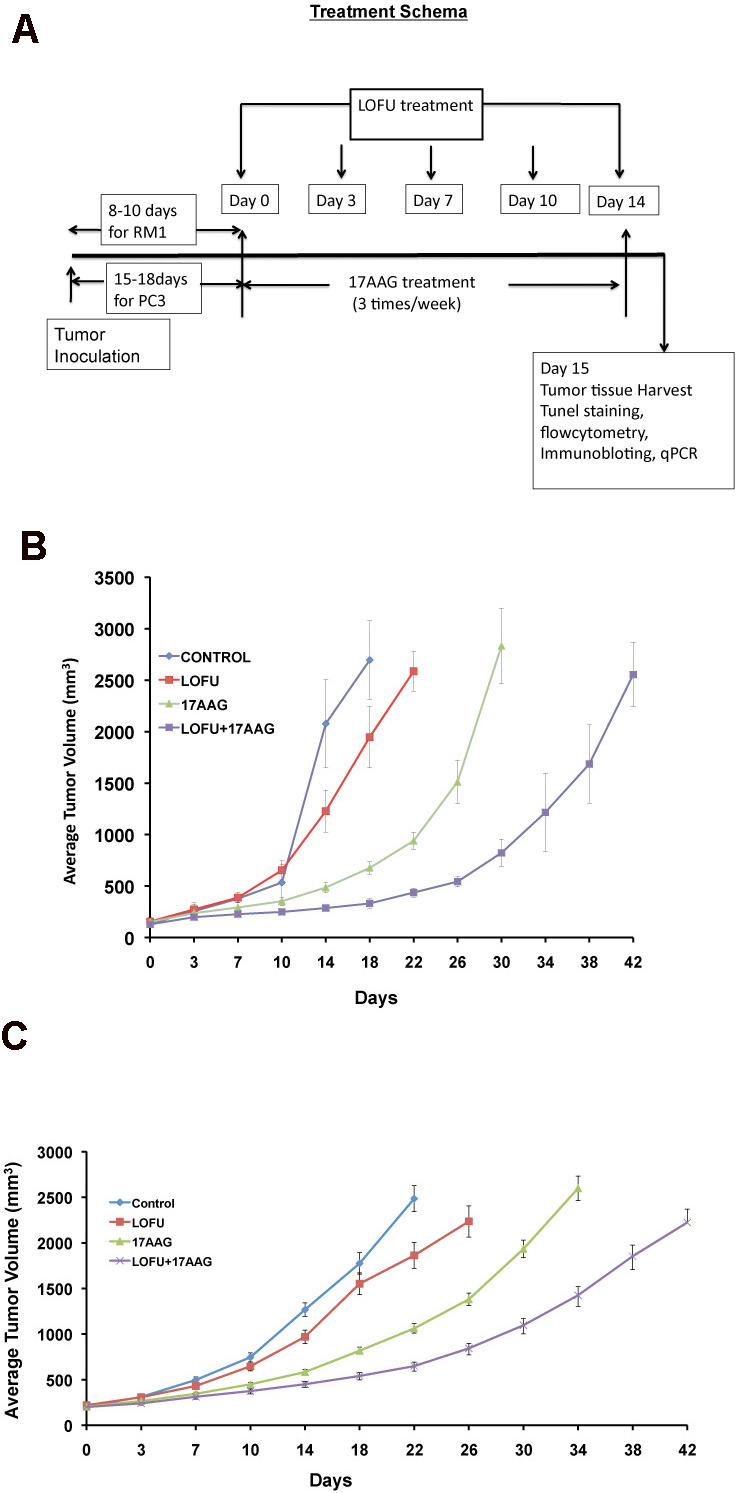
Tumor growth retardation of murine and human prostate tumors after LOFU+17AAG treatment A. Treatment schema. Palpable tumors were treated with LOFU every 3-4 days for five fractions administered over two weeks. Animals received 17AAG three times a week during this time. Tumors were harvested 24 hours after the last fraction of LOFU. B. RM1 tumor. In C57Bl6 mice, LOFU+17AAG combination treatment reduced RM1 tumor growth significantly (p<0.004), compared to controls. Note that either LOFU or 17AAG alone failed to control tumors significantly. LOFU sensitized the effects of a low dose (25mg/kg of body weight) 17AAG. C. PC3 tumor. In BalbC nu/nu mice LOFU+17AAG combination treatment showed significant reduction in PC3 tumor growth (p<0.007).

Degradation of misfolded proteins is mediated by the proteosomal pathway and autophagy. Autophagy has been implicated in the tumorigenesis process in a context-dependent role, where it might provide amino acids and other essential nutrients to the metabolic pathways of hypoxic tumors that are nutrient deprived [22]. Indeed, an increase in CMA activity has been described in a wide variety of human tumors and CMA has been implicated in survival, proliferation, and metastases of tumor cells [23]. Therefore, we quantitated the levels of two key proteins participating in autophagy, Beclin, a marker of macroautophagy, and LAMP-2A lysosomal receptor, a marker of CMA in the tumor tissues of various treatment cohorts. As shown in Figure 5, Beclin levels remain unchanged with LOFU or 17AAG or the combination therapy (Figure 4A & C), indicating that macroautophagy was not altered with ultrasound therapy. However, LOFU alone or 17AAG alone induced the expression of LAMP-2A (Figure 4A & B), indicating a compensatory increase in CMA after therapies that increase the burden of misfolded proteins in the ER. Interestingly, the combination of LOFU+17AAG inhibited the levels of LAMP-2A below the basal levels seen in these tumors. This suggests that the combination therapy reduces the growth of tumor cells and induces apoptosis by increasing ER stress while suppressing CMA.

### LOFU sensitizes human and murine prostate cancer grafts to non-toxic low doses of 17AAG

Treatment with LOFU alone or low dose of 17AAG (25 mg/kg body weight) alone did not show any normal tissue toxic effect but failed to inhibit tumor growth. However, combination therapy of LOFU+17AAG reduced the growth of murine RM1 tumors (Figure 5B). The average estimated tumor growth is 5% (p<0.0001), 9% (p<0.0001) and 11% (p<0.0001) slower in LOFU, 17AAG and LOFU+17AAG cohort compared to control group. The median time to achieve tumor size 2000 mm^3^ in control, LOFU, and LOFU+17AAG were 18, 22, and 42 days, respectively. All the animals in 17AAG group achieved the size within the interval of 26-30 days.

A similar degree of chemosensitization was observed in human PC3 tumors in BalbC nu/nu mice upon application of LOFU together with low non-toxic dose of 17AAG (14mg/kg of body weight), achieving significant tumor growth retardation (p<0.007) (Figure 5C) without any immediate adverse side effects.

### LOFU+17AAG treatment reduces the prostate cancer stem cell population in tumor tissue

**Figure 6:**
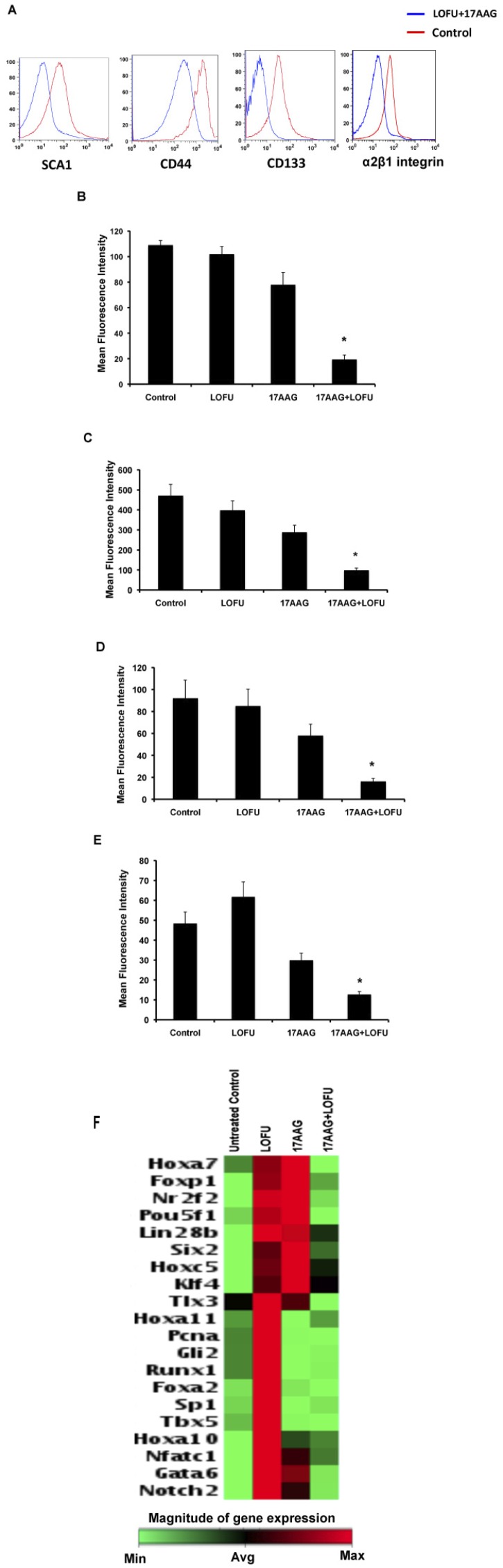
LOFU+17AAG treatment reduces the expression of prostate cancer stem cell markers in RM1 cells Flow cytometry of isolated RM1 tumor cells showed significant decrease in SCA1 (A & B), CD44 (A & C), CD133 (A & D), and α2β1 integrin (A & E) cell surface expression on RM1 tumor cells after LOFU+17AAG treatment. (F) qRT-PCR array followed by heat map analysis showed that LOFU+17AAG combination treatment group down-regulates the mRNA levels of stem cell transcription factors.

The effect of LOFU+17AAG-induced ER stress on PC stem/progenitor population was evaluated by flow cytometric analysis of PC stem/progenitor cell surface markers [24, 25]. The percentage of cells expressing cell surface SCA1 (Figure 6 A & B) (p<0.004), CD44 (Figure 6A & C) (p<0.003), CD133 (Figure 6A & D) (p<0.007), and α2β1 integrin (p<0.005) (Figure 6A & E) was significantly decreased in the combination treatment group, compared to control or single treatment cohort. Mean fluorescence intensity (MFI) of all these markers remained unaltered in all the three groups. qRT-PCR array of stem cell transcription factors demonstrated increase (>2 folds) in mRNA levels of TIx3, Hoxa11, Pcna, Gli2, Runx1, Foxa2, Sp1, Tbx5, Hoxa10, Nfatc1, Gata6, and Notch2 (Figure 6F), indicating that LOFU induces a PC stem cell transcription signaling. Treatment with 17AAG also increased the expression of some transcription factor mRNAs, such as FoxP1, Nrf2f, and Pou5f1 that were present in LOFU-treated tumors. However, tumor treated with LOFU+17AAG down-regulated the expression of these genes, suggesting that maximization of ER stress by the combination treatment might reduce the PC stem/progenitor cell population in tumors.

## DISCUSSION

In this report we demonstrate that two safe, non-toxic treatment modalities can be combined to cause significant synthetic lethality in prostate tumors. By utilizing low intensity, low energy focused ultrasound, we have significantly improved the safety of the procedure and demonstrate for the first time that focused LOFU can induce ER stress and UPR in mouse and human prostate tumors, without killing the tumor. LOFU alone did not induce apoptosis in RM1 cells, but it did induce the cytoprotective branch of UPR by augmenting the IRE1α transcript and inducing XBP1 splicing, a surrogate marker of the protective branch of UPR [19, 26]. In order to increase the tumoricidal effects of LOFU, we combined LOFU with a low dose of 17AAG, an HSP90 inhibitor. 17AAG is associated with significant hematopoeitic and hepatic toxicity that can result in mortality (Figure 1A). We adjusted the dose so that the systemic toxicity is avoided [11], but this low-dose was not effective in controlling tumors (Figure 5). However, the combination of LOFU and low-dose 17AAG reduced the XBP1 splicing, suggesting a possible inhibitory effect on pro-survival pathways of UPR. LOFU+17AAG induced the phosphorylation of PERK and eIF2α, increased ER stress and the expression of pro-apoptotic CHOP. Activation of PERK can independently shift the balance of the cell fate towards apoptosis and reduce the cytoprotective role of UPR in tumors [27, 28]. In agreement with these findings, our results demonstrate that the combination therapy reprograms the expression of pro-apoptotic genes in tumors and induces massive apoptosis in tumor xenografts, resulting in significant tumor growth retardation of mouse and human PC tumors.

In vitro, our LOFU protocol increased the temperature by 3.2 °C on average. We understand that measuring temperature using a thermocouple is not optimal monitoring because of uncertainty in the location of the thermocouple tip with respect to the focal point, and due to potential viscous heating of the thermocouple itself. Furthermore, temperature elevations in a tissue-mimicking phantom do not fully correspond to those *in vivo* during therapy due to lack of perfusion in phantom. Thus, three dimensional temperature monitoring is planned for future *in vivo* studies using, e.g., MRI-based temperature mapping. LOFU treatment, as devised in this study, is different than hyperthermia treatment because we pulse LOFU for 1.5 seconds in the focal point, while hyperthermia requires elevation of temperature for 30-90 minutes. Thus, there were no signs of normal tissue toxicity such as alopecia, thermal or mechanical damage, or skin wounds post-LOFU treatment. These observations are in agreement with the low intra-tumoral acoustic pressure, acoustic intensity, and temperature elevation, which are well below the thresholds for acoustic cavitation and thermal ablation [29, 30, 31].

High-intensity focused ultrasound (HIFU) has been used as non-invasive ablative therapy for prostate cancer [32, 33] and other solid tumors [34, 35], either with or without imaging guidance. HIFU has also been used as an effective salvage therapy for local relapse of PC after external beam radiation therapy [36]. However, a high rate of recto-urethral fistulae was reported in patients receiving trans-rectal HIFU therapy, indicating that HIFU-induced near field heating of the rectal wall could contribute to serious adverse events [32]. This is problematic because approximately 25% to 60% of patients will demonstrate biochemical recurrence following local PC treatment [37, 38]. Transurethral ultrasound therapy may have the potential to provide a greater safety profile, while enabling targeted focal, regional, or whole-gland therapy of prostate cancer ([2]). Currently, however, there are no curative treatments for recurrent and metastatic PC.

Our study is highly significant as it demonstrates that two safe therapy modalities, LOFU and low-dose 17AAG, when combined, could result in significant PC cell death and increase the therapeutic ratio for tumor control. By avoiding the high acoustic intensity and high-energy requirements for tumor ablation, LOFU would increase the safety of ultrasound therapy in these patients. Sensitization of LOFU and 17AAG is mutual, as these treatments as a single modality, although safe, are not effective in tumor control. This report, therefore, describes for the first time a new paradigm where targeted LOFU can increase ER stress and UPR in the hypoxic and nutritionally deprived tumors, while a systemic therapy with a chemotherapeutic agent, such as, 17AAG that inhibits the molecular chaperones could tip the balance from a cytoprotective arm of UPR to the PERK-CHOP-induced apoptotic arm. This combined synthetic lethality is reminiscent of other drug interactions that have been described in cancer drug screens recently, such as the use of PARP inhibitors in tumors that have BRCA1/2 deficiency [39]. ER stress in tumors has been associated with an increase in resistance to topoisomerase-II inhibitors, such as, etoposide [40], while synergizing with cisplatin [41] and other DNA crosslinking agents for tumoricidal effects [8]. Thus, LOFU-induced activation of ER stress and UPR could differentially modulate the chemosensitivity of tumors. Our results suggest that LOFU could sensitize tumors to agents that increase the ER stress in tumors. Such agents could inhibit molecular chaperones or inhibit proteosomal activity, thereby suppressing protein refolding or reducing the breakdown of misfolded proteins, respectively.

The combination of LOFU+17AAG decreased the expression of lysosomal receptor, LAMP-2A, a surrogate marker of CMA without impacting Beclin expression, a marker of macroautophagy. This is particularly interesting as CMA has been associated with tumor growth, progression, and metastatic potential of solid tumors [23, 42]. Kon et al. demonstrated that selectively knocking down the expression of LAMP-2A by shRNAs in human lung cancer cell lines resulted in a reduction of tumor xenograft growth and development of lung metastases [23]. While the use of shRNA was a proof-of-principle study, these findings may help to translate the combination of LOFU and low-dose 17AAG to clinic for the treatment of recurrent solid tumors.

Finally, LOFU+17AAG reduced the percentage of PC cells expressing PC stem/progenitor cell surface markers, e.g., CD133, CD44, Sca1, and α2β1 integrin. Whether this is due to increased sensitivity or increase in differentiation of PC stem/progenitor cells to the ER stress generated by the combination therapy is unclear. It is interesting that LOFU+17AAG down-regulated the expression of mRNAs of stem cell transcription factors in the PC tumor. Since cancer stem cells are known to be major targets for resistance to chemotherapy and radiation therapy [43-46], the combination of LOFU+17AAG could provide an opportunity of cure for recurrent PC by eliminating the PC stem cell population.

In summary, these experiments suggests a new paradigm where the combination therapy utilizing non-ablative and non-invasive LOFU and a non-toxic, low dose 17AAG increases ER stress and activates PERK-CHOP pathway of UPR. This results in the induction of apoptosis of the tumor cells and producing significant tumor growth retardation of murine RM1 and human PC3 prostate cancer xenografts in mice. This is highly significant because as individual treatment regimens, LOFU and low-dose 17AAG are safe but did not demonstrate meaningful tumoricidal effects. In addition, the combination treatment inhibited the expression of LAMP-2A, a key player in chaperone-mediated autophagy with tumor promoting functions. Thus, a novel therapy of LOFU-induced chemosensitization may be designed for locally advanced and recurrent tumors. Even in patients with systemic disease, one could envision use of LOFU to sensitize large macroscopic tumors to chemotherapy while the microscopic metastatic disease can be eradicated by chemotherapy.

## Materials and Methods

### Animals

Five- to six weeks-old male C57Bl/6 (NCI-Fort Dietrich, MD, USA) mice and athymic nude (BalbC nu/nu mice, Jackson Laboratory, Bay Harbor, ME, USA) mice were maintained *ad libitum* and all studies were performed under the guidelines and protocols of the Institutional Animal Care and Use Committee of the Albert Einstein College of Medicine.

### Tumor model and treatment

C57Bl/6 and BalbC nu/nu mice were injected subcutaneously with 1×10^5^ RM-1 (murine prostate cancer cell line) and 1×10^6^ PC3 (human prostate cancer cell line) cells on the flank, respectively. Approximately 10 days later, the tumor became palpable (3-5 mm in diameter), whereupon LOFU treatment was initiated. Mice were divided into 4 groups (n=5/group) receiving no treatment, LOFU, 17AAG (InvivoGen, San Diego, CA, USA), and 17AAG+LOFU. Palpable tumors were treated with LOFU every 3-4 days for five fractions administered over two weeks. Animals received 17AAG three times a week during this time. Tumor volume measurements were performed twice weekly using Vernier calipers along with simultaneous physical assessment of signs of systemic toxicity (malaise and diarrhea).

### LOFU system

A therapy and imaging probe system (TIPS, Philips Research North America, Briarcliff Manor, NY, USA) was utilized for all ultrasound exposures. The system includes an 8-element spherical shell annular array transducer (80 mm radius of curvature, 80 mm aperture), as well as a motion stage to allow for transducer movement and accurate positioning. The transducer was operated at 1.0 MHz, resulting in a focal spot approximately 1.5 mm in diameter and 12 mm in length (-6 dB of pressure). [12, 13])

### LOFU treatment protocol

On treatment day, the animals were anesthetized with ketamine and xylazine (7:1 mg/ml for 100 l/mouse, i.p.). Once positioned for therapy, the tumor was acoustically coupled to the TIPS system using degassed water and ultrasound gel.

Ultrasound exposure parameters were as follows: acoustic power of 3 W and a duty cycle of 100%, yielding an approximate *in situ* spatial-peak temporal-average intensity (I_spta_)[14] of 270 W/cm^2^ at a sonication depth of 3 mm in tissue, assuming an attenuation coefficient of 0.5 dB cm^−1^ MHz^−1^ [15]. Ultrasound exposures were delivered to the tumor using a 2 mm grid pattern extending over the entire tumor volume. Prior to LOFU, the tumor volume was measured to calculate the grid size for the particular treatment. The duration of LOFU exposure at each grid point was 1.5 s, after which the transducer was automatically positioned over the next grid point and the procedure repeated until the entire tumor volume was covered. This yielded a non-uniform energy delivery to the tumor.

### *In vitro* temperature rise estimation

Estimation of intra-tumoral temperature by invasive means could modulate the therapeutic response of the combination treatment. Therefore, to estimate intra-tumoral temperature elevation using the above described setup and therapy protocol, the ultrasound exposures were performed in a 6 mm × 6 mm area within a tissue-mimicking phantom, [16] into which a T-type thermocouple (diameter 200 μm) was embedded at a depth of 3 mm. These *in vitro* exposures were repeated 5 times and the results averaged.

### Detection of Apoptosis *In Situ*

Apoptotic cells were detected *in situ* by performing TUNEL (TdT–mediated digoxigenin labeled dUTP nick end labeling) staining. Briefly, paraffin embedded sections were de-paraffinized, rehydrated through graded alcohols, and stained using an ApopTag kit (Intregen Co, Norcross, GA, USA). The apoptotic rate in tumor cells was quantified by counting the percent of apoptotic cells in each high power field.

### Immunoblot Analysis

24 hr post-LOFU the tumor cells were harvested, washed with phosphate-buffered saline, and lysed using TPER (Thermo Fisher Scientific, Rockford, IL, USA). Cell lysates were subjected to SDS-PAGE, transferred to polyvinylidene difluoride membrane, and immunoblotted with primary antibodies against PERK, pPERK, eIF2, peIF2, ERp72, ERp44, ERp57, Beclin (Cell signaling, Danvers, MA, USA), Lamp2a (Abcam, Cambridge, MA, USA), and horseradish per-oxidase-conjugated secondary antibody. The blots were developed using the ECL kit (GE Healthcare, Piscataway, NJ, USA). Densitometric analysis of immunoreactive bands of each blot was photographed and then images were digitized and analyzed by using Gel Doc XR system (Bio-Rad, Hercules, CA, USA).

### Real Time PCR analysis of UPR target genes

24 hr after LOFU treatment the RM1 tumor cells were lysed using RLT buffer mixed with 1% betamercaptoethanol from RNeasy Mini Kit (Qiagen, Valencia, CA, USA). Qiagen's protocol for the RNeasy Mini Kit with on-column DNA digestion was used to isolate RNA from the tumor lysates. The RNA samples were stored at −80°C, prior to further use. Isolated RNA was subjected to cDNA synthesis using the SuperScriptTM First-Strand Synthesis System (Invitrogen, Grand Island, NY, USA). The splicing of XBP1 RNA was detected using the following primer pair 5′-ACTCGGTCTGGAAATCTG-3′ and 5′-TAGCCAGGAAACGTCTAC-3′ (Fisher Scientific, Pittsburg, PA, USA) [7]. Real time PCR was performed in Light Cycler real time PCR machine (Bio Rad Laboratories, Hercules, CA, USA) using the ABsolute QPCR SYBER Green Mix (ABgene, Rochester, NY, USA) according to the standard ABgene protocol. To check for primer amplification specificity, a melting curve was generated at the end of the PCR and different samples containing the same primer pair showed matching amplicon melting temperatures. Primers used for real time PCR included GRP78 5′TTGCTTATGGCC TGGATAAGAGGG3′ 3′ and 5′TGTACCCTTGTCTTCAGCTGTCAC3′; EDEM 5′ TCATCCGAG TTCCAGAAAGCAGTC 3′ and 5′ TTGACATAGAGTGGAGGGTCTCCT 3′ (Fisher Scientific). All the qRT-PCR and Real time PCR experiments were repeated three times. The qRT-PCR and PCR array for apoptosis genes and stem cell transcription factor were performed by SA Biosciences PCR array system (Frederick, MD, USA) according to manufacturer protocol. In brief, cDNA were prepared from purified total RNA using RT^2^ First Strand Kit (Qiagen) followed by PCR array using SA Bioscience PCR array kit. Data was analyzed by web based PCR array data analysis software from SA Biosciences.

### Flowcytometric analysis

Flank tumors were treated with LOFU, 17AAG, and LOFU+17AAG in various cohorts. 24 hours after treatment, tumor cells were isolated by collagenase digestion and analyzed by flowcytometry for the expression of prostate cancer stem cell markers, SCA1, CD44, and CD133. Isolated tumor cells were stained with anti-SCA1 conjugated with FITC (BD Biosciences, La Jolla, CA, USA), anti-CD133 conjugated with pacific blue (eBioscience, San Diego, CA, USA) and anti-CD44 conjugated with PE (BD Biosciences, La Jolla, CA, USA). Data acquisition was performed using LSRII (BD Biosciences) and analyzed by FlowJo v.7.1 (Treestar Inc, Ashland, OR, USA) software.

### Kaplan-Meier Survival analysis

Mice survival/mortality in different treatment groups was analyzed by Kaplan-Meier as a function of radiation dose using Sigma–Plot and GraphPad Prism (version 4.0 for OS X, San Diego, CA, USA) software.

### Statistical Analysis

For digital images, sampling regions were chosen at random for digital acquisition for data quantitation. Digital image data was evaluated in a blinded fashion as to any treatment. A two-tailed Student's t-test was used to determine significant differences (p<0.05) between experimental cohorts with representative standard errors of the mean (SEM).
